# Postoperative renal morbidity and mortality after volume replacement with hydroxyethyl starch 130/0.4 or albumin during surgery: a propensity score-matched study

**DOI:** 10.1007/s00540-020-02838-z

**Published:** 2020-08-11

**Authors:** Hideki Miyao, Yoshifumi Kotake

**Affiliations:** 1 Department of Anesthesiology, Saitama Medical Center, Saitama Medical University, 1981, Kamoda, Kawagoe, Saitama 350-8550 Japan; 2 Department of Anesthesiology, Toho University Ohashi Medical Center, 2-22-36, Ohashi, Meguro-ku, Tokyo, 153-8515 Japan

**Keywords:** Hydroxyethyl starch derivatives, Albumin, Propensity score, Acute kidney injury

## Abstract

**Purpose:**

We aimed to compare retrospectively the rates of renal morbidity and mortality in surgical patients receiving 6% HES 130/0.4 to those receiving albumin.

**Methods:**

From a Japanese nationwide medical database between 2014 and 2016, we identified adults who received HES 130/0.4 (HES group) or albumin (albumin group) as a single colloid solution on the day of surgery. After propensity score matching, the two groups were analyzed with *χ*^2^ or Mann Whitney *U* test. The primary outcome was the incidence of acute kidney injury (AKI). Secondary outcomes included the incidence of renal-replacement therapy, hospital length of stay, in-hospital 30-day mortality, the use of vasoactive agents, and the fluid requirement on the day of surgery.

**Results:**

Of 76,048 patients in the database, propensity score matching identified 289 matched pairs. There was no statistically significant difference in the incidence of AKI between the HES and the albumin group (15.2% vs. 20.8%, respectively: *P* = 0.08)*.* The secondary outcomes did not differ between groups except the following. Median hospital stay was 5 days shorter in the HES group (18 vs. 23 days; *P* < 0.001), and the median net fluid requirement on the day of surgery was 15 mL/kg lower in the HES group (140 vs. 155 mL/kg, respectively; *P* = 0.01).

**Conclusions:**

Postoperative renal morbidity and mortality did not differ between patients receiving HES 130/0.4 and those receiving albumin. HES 130/0.4 was associated with shorter hospital stay and less fluid requirement compared to albumin. These findings support the use of 6% HES 130/0.4 for perioperative volume replacement as an alternative to albumin.

**Trial registration:**

UMIN000027896 and the date of registration was June 30, 2017 at https://www.umin.ac.jp/ctr/index-j.html.

## Introduction

Although fluid resuscitation in surgery has been debated regarding a liberal versus a restricted strategy [1–4], consensus statement of Enhanced Recovery After Surgery (ERAS) recommended a goal directed fluid therapy (GDFT) using colloids for perioperative management to avoid fluid excess and organ hypoperfusion [3]. Colloid solutions have become important to preserve intravascular volume because the required amounts are smaller than those for crystalloids [3, 5].

Studies of critically ill nonsurgical patients showed that colloids, including albumin and hydroxyethyl starch (HES), were no better and sometimes less effective than crystalloids in reducing renal morbidity [6–10]. However, in studies of surgical patients, the newest preparation of 6% HES 130/0.4 (Voluven^®^; Fresenius Kabi GmbH, Germany) was not associated with renal damage [11–16]. A recent multicenter randomized trial for high-risk abdominal surgery compared HES 130/0.4 with crystalloid using Doppler-guided GDFT and showed no evidence of renal toxicity in patients receiving HES 130/0.4 [11].

Generally, albumin is thought to be safer than artificial colloids because it is derived from human albumin, but it can also be more expensive [17], has ethical implication as a human blood product, and is associated with a higher risk of infection [18]. Additionally, a study of patients undergoing cardiac surgery using a propensity score-matching method showed that albumin was dose-dependently associated with increasing risk of acute kidney injury (AKI) [16]. In studies of children undergoing cardiac surgery [19, 20] and in adults undergoing elective cystectomy [21], renal morbidity and safety profiles of HES 130/0.4 did not differ from those of albumin. A study reported that using 6% HES 130/0.4 as an alternative to albumin could potentially reduce the amount of albumin used to treat surgical bleeding by up to 80% [22].

These findings led us to choose HES 130/0.4 as an intraoperative volume expander instead of albumin. Our report of 9000 propensity-matched pairs of surgical patients found that 6% HES 130/0.4 was not associated with a greater incidence or severity of postoperative AKI, but instead was associated with a lower incidence of renal replacement therapy (RRT) when compared to controls who did not receive HES [13]. However, some patients in both the HES group and controls received some amount of albumin, thus, we did not clearly distinguish HES 130/0.4 from albumin as a volume expander. Therefore in the same nationwide database used in our previous study [13], we compared 6% HES 130/0.4 with albumin on postoperative renal morbidity and mortality in patients undergoing various types of surgery.

## Methods

The study protocol was reviewed by the Toho University Ohashi Medical Center institutional review board (Ref: H16105), which waived formal approval and the requirement for written informed consent because the archived data were fully de-identified. The study protocol was registered with the UMIN Clinical Trial Registry of the Japanese University Hospital Medical Information Network on June 30, 2017 (https://www.umin.ac.jp/ctr/index-j.htm: registry number: UMIN000027896).

**Fig. 1:**
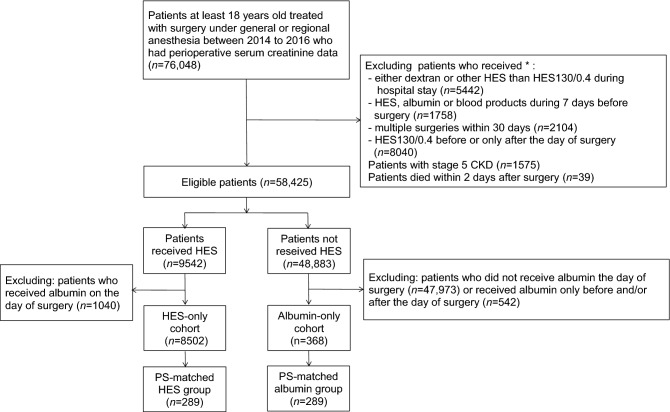
Sample selection for propensity score matching in a study comparing 6% HES 130/0.4 to albumin on postoperative renal morbidity. *CKD* chronic kidney disease; *HES* hydroxyethyl starch; *PS* propensity score. *Multiple exclusion criteria were applied because some patients met two or more exclusion criteria

This study was designed by the authors with assistance of several persons with statistical expertise from Otsuka Pharmaceutical Factory Inc. (Tokushima, Japan). The data extraction and statistical analyses were made by Medical Data Vision Corp. (Tokyo, Japan) according to our policies and procedures. Database of Medical Data Vision was extracted from the Japanese Medical Database for Healthcare Reimbursement (the Diagnosis Procedure Combination/Per-Diem Payment System; DPC/PDPS) [23]. Other information of study design, data sources, study population, and exclusion criteria are detailed in our previous report [13] and the present (Fig. 1).

### Patient assignment

Patients were assigned to the HES group if they had received any amounts of 6% HES 130/0.4, but no albumin on the day of surgery, and to the albumin group if they had received any amounts of albumin but no HES on the day of surgery. Amount of albumin concentrates was mathematically converted to the equivalent volume of 5% albumin.

### Statistical methods

Data are summarized as medians and interquartile ranges or as numbers and percentages. The covariates used for propensity score (PS) matching (Table 1), preoperative comorbidity codes (Table2), and statistical method to model receiving HES 130/0.4 and estimate the PS for each patient are the same and detailed in our previous report [13].

**Table 1:** Covariates used for propensity score matching in a study of renal morbidity comparing 6% HES 130/0.4 to albumin for volume replacement

Covariate	Level of measurement
Age, years	Continuous
Male sex	Binary
Body mass index, kg/m^2^	Continuous
Hospital capacity	Ordinal
< 200 beds	
200–499 beds	
≥ 500 beds	
Year of treatment	Ordinal
2014	
2015	
2016	
Preoperative serum creatinine, mg/dL	Continuous
Preoperative radiocontrast use	Binary
Emergency surgery	Binary
Preoperative comorbidities	
Myocardial infarction	Binary
Congestive heart failure	Binary
Peripheral arterial disease	Binary
Cerebrovascular disease	Binary
Chronic obstructive lung disease	Binary
Chronic liver disease	Binary
Portal hypertension	Binary
Ascites	Binary
Diabetes mellitus	Binary
Malignancy	Binary
Arrhythmia	Binary
Valvular heart disease	Binary
Hypertension	Binary
Chronic kidney disease	Binary
Anemia	Binary
Septicemia	Binary
Types of surgery^a^	
Cardiovascular with CPB	Binary
Cardiovascular without CPB	Binary
Open thoracic	Binary
Open gastrointestinal	Binary
Open hepatobiliary	Binary
Open orthopedic	Binary
Open gynecologic/urologic/obstetric	Binary
Craniotomy	Binary
Miscellaneous	Binary
Anesthetic management	
Anesthesia duration, minutes	Continuous
Anesthetic method	Categorical
General anesthesia	
Regional anesthesia	
General with regional anesthesia	
Transfusion on the day of surgery, mL	Ordinal
No transfusion	
1–500	
501–1000	
> 1000	

*CPB* cardiopulmonary bypass

^a^Types of surgery were counted as binary because some patients received multiple types of surgery on the day of surgery

**Table 2:** International classification of disease, 10th revision, preoperative comorbidity codes used in a study of renal morbidity comparing 6% HES 130/0.4 to albumin for volume replacement

Myocardial infarction	I21.x, I22.x, I25.2
Congestive heart failure	I09.9, I11.0, I13.0, I13.2, I25.5, I42.0, I42.5-I42.9, I43.x, I50.x, P29.0
Peripheral arterial disease	I70.x, I71.x, I73.1, I73.8, I73.9, I77.1,I79.0, I79.2, K55.1, K55.8, K55.9, Z95.8, Z95.9
Cerebrovascular disease	G45.x, G46.x, H34.0, I60.x-I69.x
Chronic obstructive lung disease	I27.8, I27.9, J40.x–J47.x, J60.x–J67.x, -J68.4, J70.1, J70.3
Chronic liver disease	B18.x, K70.0–K70.3, K70.9, K71.3–K71.5, K71.7, K73.x, K74.x,K76.0, K76.2–K76.4, K76.8, K76.9, Z94.4, I85.0, I85.9, I86.4, I98.2, K70.4, K71.1, K72.1, K72.9, K76.5, K76.6, K76.7
Portal hypertension	K766
Ascites	A183, C786, I898, N289, R18
Diabetes mellitus	E10.0, E10.1, E10.6, E10.8, E10.9,E11.0, E11.1, E11.6, E11.8, E11.9, E12.0, E12.1, E12.6, E12.8, E12.9, E13.0, E13.1, E13.6, E13.8, E13.9, E14.0, E14.1, E14.6, E14.8, E14.9, E10.2–E10.5, E10.7, E11.2–E11.5, E11.7, E12.2–E12.5, E12.7, E13.2–E13.5, E13.7, E14.2–E14.5, E14.7
Malignancy	C00.x–C26.x, C30.x–C34.x, C37.x–C41.x, C43.x, C45.x–C58.x, C60.x–C76.x, C81.x–C85.x, C88.x, C90.x–C97.x, C77.x–C80.x
Arrhythmia	I44.1–I44.3, I45.6, I45.9, I47.x–I49.x, R00.0, R00.1, R00.8, T82.1, Z45.0, Z95.0
Valvular heart disease	A52.0, I05.x–I08.x, I09.1, I09.8, I34.x–I39.x, Q23.0–Q23.3, Z95.2-Z95.4
Hypertension	I10.x, I11.x–I13.x, I15.x
Chronic kidney disease	I12.0, I13.1, N03.2-N03.7, N05.2-N05.7, N18.x, N19.x, N25.0, Z49.0-Z49.2, Z94.0, Z99.2
Anemia	D50.0, D50.8, D50.9, D51.x–D53.x
Septicemia	A02.1, A20.7, A22.7, A24.1, A26.7, A28.8. A32.7, A39.4, A40.x, A41.x, A42.7, A54.8, B00.7, B34.9, B37.7

Specifically, each patient who received HES was paired with a patient who received albumin with the ascending method (i.e., the pairing goes from the patient with the lowest PS in the HES group to the highest. The reason for selecting the ascending method is described below). A patient in the albumin group was matched with a patient in the HES group having the nearest PS and was selected if the caliper was within 0.2 standard deviations of the PS logit. The caliper range is the maximum accepted difference between patients matched on a covariate. A caliper within 0.2 standard deviations of the PS log is widely accepted as appropriate [24]. Patients who could not be matched were not included when assessing the relationship of HES or albumin to the outcomes.

To examine the balance of baseline variables between groups, the standardized difference [25] (the difference in means or proportions divided by the pooled standard deviation) was calculated before and after PS matching [26]. When the standardized difference was less than 10%, we considered the groups to be balanced on the covariate [27].

The software can perform three types of matching; descending, ascending and random. We first used the software’s default method, the descending method. After PS matching, four covariates exceeded 10% of the standardized difference: preoperative septicemia (10.8%), cardiovascular surgery with cardiopulmonary bypass (12.3%), anesthesia duration (14.0%), and transfusion on the day of surgery (15.4%), differences large enough to affect outcomes. However, the ascending method balanced the covariates in both groups, with only two exceptions: age (12.4%) and miscellaneous surgeries (10.2%; Table 3). We thought these two covariates would affect outcomes to a lesser degree than the four covariates listed above and so used the ascending method to pair patients before analyzing the outcomes to minimize selection bias in matching.

**Table 3:** Standardized differences of covariates before and after propensity score matching by the ascending method in a study of renal morbidity comparing 6% HES 130/0.4 to albumin for volume replacement

Covariate	Before PS matching			After PS matching		
	HES group (*n* = 8502)	Albumin group (*n* = 368)	Standardized difference %^a^	HES group (*n* = 289)	Albumin group (*n* = 289)	Standardized difference %^a^
Age, median (IQR), years	68 (54–76)	75 (67–81)	**62.6**	75 (68–82)	74 (66–81)	**12.4**
Male sex, *n* (%)	3962 (46.6)	218 (59.2)	**25.5**	186 (64.4%)	172 (59.5%)	10.0
BMI, median (IQR), kg/m^2^	23.0 (20.6–25.5)	22.0 (19.2–24.3)	**31.8**	21.6 (19.3–24.3)	22.0 (19.2–24.1)	3.9
Hospital capacity, *n* (%)						
< 200 beds	127 (1.5)	8 (2.2)	**70.4**	0 (0.0)	8 (2.8)	
200–499 beds	6292 (74.0)	142 (38.6)		120 (41.5)	116 (40.1)	5.1
≥ 500 beds	2083 (24.5)	218 (59.2)		169 (58.5)	165 (57.1)	
Year of treatment, *n* (%)						
2014	1172 (13.8)	108 (29.3)	**22.4**	70 (24.2)	88 (30.4)	
2015	3409 (40.1)	108 (29.3)		120 (41.5)	78 (27.0)	4.0
2016	3921 (46.1)	152 (41.3)		99 (34.3)	123 (42.6)	
Preoperative sCr, median (IQR), mg/dL	0.69 (0.56–0.84)	0.86 (0.66–1.07)	**59.6**	0.83 (0.67–1.01)	0.83 (0.63–1.03)	7.9
Received preop radiocontrast, *n* (%)	438 (5.2%)	50 (13.6%)	**29.3**	40 (13.8)	43 (14.9)	3.0
Preoperative comorbidities, *n* (%)						
Myocardial infarction	226 (2.7)	19 (5.2)	**12.9**	16 (5.5)	15 (5.2)	1.5
Congestive heart failure	788 (9.3)	137 (37.2)	**70.1**	90 (31.1)	83 (28.7)	5.3
Peripheral artery disease	689 (8.1)	75 (20.4)	**35.7**	54 (18.7)	50 (17.3)	5.4
Cerebrovascular disease	818 (9.6)	61 (16.6)	**20.7**	54 (18.7)	48 (16.6)	5.4
COPD	794 (9.3)	47 (12.8)	**11.0**	34 (11.8)	33 (11.4)	1.1
Chronic liver disease	896 (10.5)	83 (22.6)	**32.8**	69 (23.9)	68 (23.5)	0.8
Portal hypertension^b^	4 (0.0)	0 (0.0)	–	0 (0.0)	0 (0.0)	–
Ascites	266 (3.1)	20 (5.4)	**11.4**	13 (4.5)	18 (6.2)	7.7
Diabetes mellitus	2223 (26.1)	159 (43.2)	**36.4**	130 (45.0)	119 (41.2)	7.7
Malignancy	3913 (46.0)	181 (49.2)	6.3	169 (58.5)	160 (55.4)	6.3
Arrhythmia	746 (8.8)	100 (27.2)	**49.4**	63 (21.8)	64 (22.1)	0.8
Valvular heart disease	747 (8.8)	121 (32.9)	**62.1**	69 (23.9)	67 (23.2)	1.6
Hypertension	3070 (36.1)	225 (61.1)	**51.7**	164 (56.7)	166 (57.4)	1.4
Chronic kidney disease	170 (2.0)	28 (7.6)	**26.5**	21 (7.3)	17 (5.9)	5.6
Anemia	1445 (17.0)	62 (16.8)	0.4	52 (18.0)	50 (17.3)	1.8
Septicemia	142 (1.7)	27 (7.3)	**27.6**	21 (7.3)	20 (6.9)	1.3
Types of surgery^c^, *n* (%)						
Cardiovascular with CPB	66 (0.8)	107 (29.1)	**86.5**	49 (17.0)	47 (16.3)	1.9
Cardiovascular without CPB	344 (4.0)	51 (13.9)	**34.9**	40 (13.8)	42 (14.5)	2.0
Open thoracic^b^	82 (1.0)	0 (0.0)	–	10 (3.5)	0 (0.0)	–
Open gastrointestinal	1245 (14.6)	119 (32.3)	**42.7**	106 (36.7)	104 (36.0)	1.4
Open hepatobiliary	584 (6.9)	67 (18.2)	**34.8**	69 (23.9)	63 (21.8)	4.9
Open orthopedic	1961 (23.1)	24 (6.5)	**47.9**	20 (6.9)	24 (8.3)	5.2
Open gynecologic/urologic/obstetric	1742 (20.5)	11 (3.0)	**56.5**	7 (2.4)	11 (3.8)	8.0
Craniotomy	186 (2.2)	4 (1.1)	8.7	2 (0.7)	4 (1.4)	6.8
Miscellaneous	2550 (30.0)	27 (7.3)	**60.8**	19 (6.6)	27 (9.3)	**10.2**
Emergency surgery, *n* (%)	203 (2.4)	27 (7.3)	**23.2**	23 (8.0)	22 (7.6)	1.3
Anesthesia duration, median (IQR), min	214 (145–300)	306 (210–412)	**62.6**	305 (210–395)	287 (194–399)	3.7
Anesthesia technique, *n* (%)						
General anesthesia	4389 (51.6)	262 (71.2)	**48.6**	188 (65.1)	188 (65.1)	0.0
Regional anesthesia	764 (9.0)	3 (0.8)		4 (1.4)	2 (0.7)	
Both general and regional	3349 (39.4)	103 (28.0)		97 (33.6)	99 (34.3)	
Transfusion on the day of surgery, *n* (%)						
No transfusion	7657 (90.1)	146 (39.7)	**126.1**	140 (48.4)	140 (48.4)	5.4
1–500, mL	346 (4.1)	37 (10.1)		24 (8.3)	32 (11.1)	
501–1000, mL	205 (2.4)	35 (9.5)		24 (8.3)	32 (11.1)	
> 1000, mL	294 (3.5)	150 (40.8)		101 (34.9)	85 (29.4)	

*BMI* body mass index; *COPD* chronic obstructive pulmonary disease; *CPB* cardiopulmonary bypass; HES hydroxyethyl starch; *IQR* interquartile range; *sCr* serum creatinine

^a^Bold values (with standardized differences > 10%) show imbalanced characteristics

^b^Portal hypertension and open thoracic surgery were excluded from the calculation of standardized difference and propensity score, because there was no case before and after matching

^c^Standardized difference was calculated for each type of surgery because some patients underwent multiple procedures on the day of surgery

### Associations between HES or albumin and outcomes

The primary outcome was the incidence of AKI within 7 days after surgery in both groups, where AKI was defined by the serum creatinine concentration set by the Kidney Disease: Improving Global Outcomes (KDIGO) criteria [28]. Only the creatinine criterion was applied because the database did not include urine output data. Thus, stage one AKI was defined as a postoperative creatinine concentration that was 1.5–1.9 times higher than the baseline (preoperative) concentration or by an absolute increase of 0.3 mg/dL from baseline. Likewise, stage two was defined as a concentration 2.0–2.9 times higher than baseline, and stage three, as concentration three times higher than the baseline value, an absolute increase of 4.0 mg/dL from baseline, or RRT begun within 7 days after surgery.

Secondary outcomes were assessing whether HES was associated with worsening AKI stage as compared to albumin, the incidence of RRT begun within 21 days after surgery, postoperative in-hospital 30-day mortality, and length of postoperative hospital stay, the use of vasoactive agents (ephedrine, phenylephrine, dopamine, dobutamine, norepinephrine, or epinephrine), net fluid requirement on the day of surgery, and the postoperative change of hemostatic and coagulation variables [platelet count, prothrombin time-international normalized ratio (PT-INR), and activated partial thromboplastin time (APTT)] within 7 days after surgery from the preoperative baseline). Patients who died during their hospital stay were not counted in the length of hospital stay. Death after hospital discharge could not be detected because the database does not contain these data.

All outcomes were compared between groups after PS matching. Categorical variables were analyzed with *χ*^2^ tests, which is unpaired and rational in 1:1 propensity score matching [29, 30]. Continuous variables were analyzed with Mann–Whitney *U* tests because Kolmogorov–Smirnov tests found no normal distributions. To determine whether HES was associated with worsening AKI stage as compared to albumin, we performed ordinal logistic regression analysis on the ordinal variable defined as AKI stage zero (no AKI), one, two, three.

The sample size was the total number of eligible patients seen during the 3-year study period; no a priori sample size calculation was performed because the study was retrospective. In recent literature, the postoperative incidence of AKI defined by KDIGO or “Risk of renal failure, Injury to the kidney, Failure of kidney function, Loss of kidney function, and End-stage renal failure” (RIFLE) criteria in patients treated with HES 130/0.4 ranged from 5 to 10% [13, 14]. On the other hand, that with albumin defined by RIFLE or Acute Kidney Injury Network (AKIN) criteria was reported from 16 to 30% [16, 21]. We assumed, therefore, that a 10% difference in the primary outcome would be clinically relevant and calculated that 540 matched patients (270 pairs) were needed to have 80% power to detect a 10% absolute difference in the incidence of AKI between the HES and the albumin group at the 0.05 level.

Data were analyzed with the SAS software program [SAS version 9.4M6 (TS1M6), SAS Institute, Cary, NC, USA], which contains the new SAS official macro program for propensity score matching. Alpha was set at 0.05, and all tests were two-tailed.

## Results

Among the 16,870,000 patients in the database, 76,048 surgical patients were treated under general or regional anesthesia, or both, and had both pre- and postoperative data on serum creatinine concentrations. Propensity matching yielded 289 matched pairs (Fig. 1). No data were missing in the covariates, but in the matching process, no patient had portal hypertension as a preoperative comorbidity, and none underwent open thoracic surgery in the albumin group (Table 3), so these two covariates were excluded from the calculation of standardized difference and propensity score for mathematical reason. The standardized differences of the covariates before and after matching show that the markedly heterogeneous groups before matching became relatively homogeneous after matching; that is, the number of covariates with standardized differences exceeding 10% was reduced from 31 to 2 after matching (Table 3).

The two groups did not differ in the incidence of AKI (HES, 15.2% vs. albumin, 20.8%: OR, 0.69; 95% CI, 0.45–1.05), worsening stage of AKI (OR, 1.49; 95% CI, 0.97–2.28), the incidence of RRT (HES, 2.4% vs. albumin, 2.1%), or 30-day mortality (HES, 2.1% vs. albumin 4.5%). Median hospital stay was 5 days shorter in the HES group than in the albumin group (18 vs. 23 days, respectively; *P* < 0.001). Use of vasoactive agents did not differ significantly between the groups (88.9% vs. 87.5%), but the median net fluid requirement on the day of surgery was 15 mL/kg lower in the HES group (139.7 vs. 154.6 mL/kg; *P* = 0.01).

No data on exposure or outcome variables were missing except for PT-INR and APTT. Data of PT-INR and APTT were missing in 296 (51.2%) and 441 (76.3%) patients after matching and, consequently, only 77 pairs and 16 pairs, respectively, were included in the outcome analysis. The median change of platelet count (−5.6 vs. − 6.4 × 10^4^/µL), PT-INR (0.15 vs. 0.19), and APTT (8.9 vs. 7.1 s) did not differ significantly between the groups (Table 4).

**Table 4:** Outcomes before and after propensity score matching in a study of renal morbidity comparing 6% HES 130/0.4 with albumin for volume replacement

	Before PS matching		After PS matching			
Outcome	HES group (*n* = 8502)	Albumin group (*n* = 368)	HES group (*n* = 289)	Albumin group (*n* = 289)	Odds ratio (95% CI)	*P* value
AKI, *n* (%)	437 (5.1)	88 (23.9)	44 (15.2)	60 (20.8)	0.69 (0.45–1.05)	0.08
Worsening AKI stage, *n* (%)					1.49 (0.97–2.28)	0.07
Stage 0	8065 (94.9)	280 (76.1)	245 (84.8)	229 (79.2)	–	–
Stage 1	374 (4.4)	61 (16.6)	36 (12.5)	45 (15.6)	–	–
Stage 2	43 (0.5)	13 (3.5)	2 (0.7)	8 (2.8)	–	–
Stage 3	20 (0.2)	14 (3.8)	6 (2.1)	7 (2.4)	–	–
Patients on RRT, *n* (%)	11 (0.1)	15 (4.1)	7 (2.4)	6 (2.1)	1.17 (0.39–3.53)	0.78
RRT duration, *n* (% of total number of patient on RRT)						
1–27 days	10 (90.9)	14 (93.3)	6 (85.7)	6 (100.0)	–	–
28–89 days	1 (9.1)	1 (6.7)	1 (14.3)	0 (0.0)	–	–
≥ 90 days	0 (0.0)	0 (0.0)	–	–	–	–
Postoperative hospital stay (days), median (IQR)^a^	11 (8–20)	23 (15–37)	18 (13–27)	23(14–38)	–	<0.001
In-hospital 30-day mortality *n* (%)^b^	32 (0.4)	16 (4.3)	6 (2.1)	13 (4.5)	0.45 (0.17–1.20)	0.11
Use of any vasoactive agent, *n* (%)	6795 (79.9)	330 (89.7)	257 (88.9)	253 (87.5)	1.14 (0.69–1.90)	0.61
Ephedrine	5056 (59.5)	214 (58.2)	173 (59.9)	164 (56.7)	–	–
Phenylephrine	4561 (53.6)	266 (72.3)	185 (64.0)	194 (67.1)	–	–
Dopamine	971 (11.4)	104 (28.3)	66 (22.8)	73 (25.3)	–	–
Dobutamine	146 (1.7)	101 (27.4)	53 (18.3)	51 (17.6)	–	–
Norepinephrine	262 (3.1)	125 (34.0)	75 (26.0)	73 (25.3)	–	–
Epinephrine	815 (9.6)	33 (9.0)	12 (4.2)	26 (9.0)	–	–
Fluid summary mL/kg per patient, median (IQR)						
Net fluid on the day of surgery	86.0 (65–117)	172.1 (119–240)	139.7 (101–192)	154.6 (109–221)	–	0.01
Crystalloid	72.6 (53–103)	157.9 (112–226)	125.7 (87–174)	140.4 (100–199)	–	–
HES 130/0.4	10.7 (8.3–16.7)	–	13.2 (9.3–19.9)	–	–	–
Albumin	–	11.0 (7.4–16.5)	–	10.4 (6.9–16.1)	–	–
Changes of hemostasis and coagulation variables						
Δ Platelet count, median (IQR) x10^4^/μL	−3.4 (−5.9 to −1.2)	−6.9 (−11.7 to −3.3)	−5.6 (−8.6 to −3.0)	−6.4 (−11.0 to −3.0)	–	0.11
Δ PT-INR, median (IQR)	0.11 (0.05–0.19)	0.21 (0.09–0.42)	0.15 (0.03–0.28)	0.19 (0.08−0.38)	–	0.43
Sample size^d^	1210	209	77	77	–	
Δ APTT, median (IQR) second	4.3 (0.7–8.2)	5.1 (0.8–12.35)	8.9 (2.2–22.4)	7.1 (0.8–5.0)	–	0.57
Sample size^d^	671	84	16	16	–	

*PS* propensity score, *AKI* acute kidney injury; *HES* hydroxyethyl starch 130/0.4; *RRT* renal-replacement therapy, *PT-INR* prothrombin time-international normalized ratio; *APTT* activated partial thromboplastin time; Δ change from preoperative value to postoperative one

^a^The postoperative days of patients who died in the charged hospital were not counted in the length of postoperative hospital stay

^b^Death after hospital discharge was not counted in mortality rate

^c^5% albumin equivalent

^d^Preoperative or postoperative data were not recorded in some patients

## Discussion

In this retrospective cohort study with 289 matched pairs of surgical patients, the incidence of AKI and worsening AKI stage in patients receiving HES did not differ significantly from those receiving albumin. Likewise, in-hospital 30-day mortality did not differ between the groups. The changes in platelet count, PT-INR, and APTT did not differ between the groups, but sample sizes of the latter two were not large enough to evaluate statistically. The only differences in outcomes were that median hospital stay was 5 days shorter and that median net fluid requirements on the day of surgery was 15 mL/kg less in patients receiving 6% HES 130/0.4.

Among many studies focused on crystalloid versus colloid therapy, the Colloids Versus Crystalloids for the Resuscitation of the Critically Ill (CRISTAL) study (*N* = 2857) showed that colloids, including albumin, HES, and gelatins, provided better outcomes than crystalloid given to patients in hypovolemic shock. That is, colloids were associated with longer mechanical ventilator-free days, vasopressor-free days, and lower 90-day mortality than crystalloids, although the primary outcome, 28-day mortality, did not differ between the groups [31]. A retrospective cohort study (*N* = 1,051,441 patients) undergoing elective total hip and knee arthroplasties showed that 6% Hetastarch (HES 450/0.7), which is the first-generation HES, and 5% albumin were associated with an increased risk of acute renal failure compared to crystalloid [32]. On the other hand, in a retrospective cohort study of patients undergoing on-pump cardiac surgery, use of both albumin and 10% pentastarch (HES 250/0.45, the second generation of HES) was associated with a dose-dependent risk for AKI. This same study showed that no dose-dependent risk for AKI was seen in patients receiving the third-generation HES 130/0.4 [16]. A meta-analysis on the safety of modern tetrastarches in surgery (the third generation of HES 130/0.4 and HES 130/0.42: 59 studies, 4529 pooled patients) concluded that “there were no indications that the use of tetrastarches during surgery induces adverse renal effects as assessed by change or absolute concentrations of serum creatinine or need for renal replacement therapy” [15]. Also, in two randomized controlled trials [19, 33] and a retrospective cohort study [20] of children undergoing cardiac surgery, HES 130/0.4 was associated with less fluid balance and lower amounts of transfusion than albumin and was not negatively associated with postoperative outcomes, including renal morbidity. A randomized controlled trial in patients undergoing cystectomy found that perioperative changes in cystatin C as a component of glomerular filtration rate and neutrophil gelatinase-associated lipocalin as a marker of tubular injury did not differ between balanced HES 130/0.4 (Volulyte^®^; Fresenius Kabi GmbH, Germany) and albumin [21]. All of the literature above support the use of 6% HES 130/0.4 as an effective and safe alternative to albumin during surgery.

### Recent fluid strategy in surgery

Many reports confirm the value of intraoperative restricted fluid therapy [1, 2, 34–36]. However, in the Restrictive versus Liberal Fluid Therapy for Major Abdominal Surgery (RELIEF) randomized trial of 3000 surgical patients, the incidence of AKI, RRT, and surgical site infection was higher in those receiving intraoperatively restricted fluids (median, 6.5 mL/kg/h) than in those receiving a liberal amount of fluid (10.9 mL/kg/h) with crystalloids and colloids including albumin and HES [4]. A review of perioperative fluid therapy for major surgery [37] and ERAS statement [3] recommended GDFT for patients with high-risk comorbidities and those undergoing high-risk surgery. Most studies of GDFT have used colloids for volume expansion [38]. A recent study showed that postoperative complications were lower when using balanced HES 130/0.4 than when using crystalloids for resuscitation in a closed-loop system [39]. An animal study [40] and a clinical study [41] found that HES 130/0.4 maintained higher level of oxygenation in the renal medulla than crystalloid. Thus, recent fluid therapy in surgery has had a GDFT strategy with colloids to avoid administering either too much or too little fluid [3, 37]. That is, the use of artificial colloids is an important component of recent fluid strategy in surgery.

### Conserving albumin

Judging from our data before PS matching, albumin, when compared to HES 130/0.4, was used in older patients with higher preoperative creatinine concentrations, more preoperative comorbidities, higher-risk surgeries, and in longer anesthesia time (Table 3). Albumin products are potentially contaminated by microorganisms [18], have some ethical issues because they are human blood products and cost more than artificial colloids [17]. As the high amounts of albumin consumption has been a problem in Japan, the Safety Committee of the Japanese Society of Anesthesiologists published a report with regard to conserving albumin and stated that if HES 130/0.4 had been used as an alternative to albumin, the amount of albumin used to treat surgical bleeding could have been reduced by up to 80% [22].

### Implication of the results

There is a difference in the range of AKI incidence between our previous report (5.6–6.2%) and the present study (15.2–20.8%) [13]. The same differences are seen in RRT incidence (0.2–0.4 vs. 2.1–2.4%), hospital stay (11–12 vs. 18–23 days), and 30-day mortality (0.5–0.6 vs. 2.1–4.5%). These findings indicate that the risk of patient background or surgical risk would be greater in the present study than those in our previous report. Anesthesiologist would preferentially choose albumin, HES, or crystalloid as a volume expander in this order for high-risk patients, highly invasive, hemorrhagic, and complex procedures. This tendency can be seen in the lower-risk proportion of patients in the HES group to that in the albumin group (before PS matching in Table 3) and in the proportion vice vasa in our previous report. Therefore, as a patient population using HES or albumin has inevitable, intrinsic, and unbalanced characteristics, it is difficult to control this bias for the large-scale study comparing HES, albumin, and crystalloid such as a study for over a one million patient population [32]. The present propensity score matching balanced both demographic and surgical characteristics between the two groups. However, although propensity score matching mimics some of the characteristics of a randomized trial, it does not allow the same control over bias and confounding [42].

Median hospital stay was 5 days shorter and the median net fluid requirement on the day of surgery was 15 mL/kg less in the HES group than those in the albumin group, which lowered costs and improved patient care, in addition to adhering to the recent trend to avoid excess fluid. In our previous report, by contrast, in which patients with HES and those without HES were compared, hospital stay was 1 day longer and fluid requirement was 14 mL/kg greater in the HES group [13]. The primary explanation for the present results is that the shorter hospital stay and less fluid requirement could be the result of the intrinsic effect of 6% HES 130/0.4. In our previous report, we discussed Hodges–Lehman median difference for the hospital stay and “unbalanced surgical invasiveness”. Hodges–Lehman median difference, the robust and unbiased estimator, indicated no difference in the hospital stay between groups. We also discussed that the greater fluid requirement would be the result of greater surgical invasiveness in the HES group, which was recognized with greater transfusion requirement and more frequent use of vasoactive agent in the HES group compared to no-HES controls [13]. In the present study, the surgical invasiveness would be fairly balanced after PS matching, which is recognized with equivalent transfusion requirement and use of vasoactive agent between groups (Tables 3, 4). Therefore, the unbalanced surgical invasiveness could not explain the contrast results. Another explanation for the contrast results is that some bias like unmeasured different patient background or unrecognized unbalance of surgical invasiveness still existed between groups after PS matching, which was discussed in the former paragraph. Such biases might partially affect the contrast results.

The present study did not clearly demonstrate the safety profile of 6% HES 130/0.4 compared to albumin, but did present its advantageous profile as a volume expander in higher-risk patient population rather than in the previous study. Although these findings are meaningful, further study is warranted to validate the findings.

### Strengths and limitations of the study

Although the 289 matched pairs have a large enough statistical power (over 80%) to detect a 10% absolute difference in the primary outcome at the 0.05 level, the actual AKI incidence was 5.6% lower in the HES group than that in the albumin group. Then, our sample size had only 42% power to detect a significant difference in primary outcome. If we had obtained a larger sample size such as over 500 pairs, the primary outcome might have achieved statistical significance.

Intraoperative hypotension and blood loss are the main risks for AKI, but the database we used does not contain these data. Low urine output is also one of the global outcomes criteria for AKI, but again, the database does not contain these data. The absence of data on blood pressure, blood loss, and urine output could have affected our results. The number of pairs matching hemostasis and coagulation system variables was small (but not for platelet count), so the changes in PT-INR (*n* = 77 pairs) and APTT (*n* = 16 pairs) could not be properly evaluated.

Among many studies reporting no negative safety profile of HES 130/0.4 in each unique type of surgery [12, 14, 16, 19–21, 33], our study covering the entire types of surgery would have a greater generalizability. The manufacturer and distributor of HES 130/0.4 in Japan funded this study. Although we believe that our study was unbiased by this fact, this potential conflict of interest should be considered as a possible bias as we mentioned in our previous study [13].

## Conclusions

In this propensity-matched study, the incidence and severity of postoperative acute kidney injury, the incidence of renal-replacement therapy, 30-day mortality, and the change of postoperative platelet count did not differ between patients receiving 6% HES 130/0.4 and those receiving albumin on the day of surgery. The length of hospital stay was shorter, and the net fluid requirement on the day of surgery was lower in the HES group. These findings support the use of 6% HES 130/0.4 as an alternative colloid to albumin for perioperative volume replacement.
